# Factors influencing institutional delivery service utilization in Dembecha district, Northwest Ethiopia: A community based cross sectional study

**DOI:** 10.1186/s12978-017-0359-5

**Published:** 2017-08-22

**Authors:** Sewnet Kidanu, Genet Degu, Tenaw Yimer Tiruye

**Affiliations:** 1 Dembecha district, Dembecha, Ethiopia; 2 College of Health Science, Debre Markos University, PO Box: 269, Debre Markos, Ethiopia

**Keywords:** Institutional delivery, Less than two years, Associated factors, Dembecha, Northwest Ethiopia

## Abstract

**Background:**

The risk of death from complications relating to pregnancy and childbirth over the course of a woman’s lifetime is higher in the developing countries. Improving the health of mothers and children through well-organized institutional delivery service is central to achieve reduced maternal and child morbidity and mortality. So, factors that underlie the level of institutional delivery service utilization need to be investigated, especially in areas where little is known about the problem. Therefore, the objective of this study was to assess factors influencing institutional delivery service utilization in Dembecha district, Northwest Ethiopia.

**Methods:**

Community based quantitative cross-sectional study was conducted from March 1 to 30, 2015 among 674 mothers who gave birth within the last two years preceding the study using interviewer administered questionnaire. Multi-stage sampling with stratification sampling technique was used. Descriptive statistics were done to characterize the study population using different variables. Bivariate and multivariable logistic regression models were fitted to determine association. Odds ratios with 95% confidence intervals were computed. Statistical significance was declared at *p*-value <0.05.

**Results:**

Of all 674 respondents, 229(34%, 95% CI: 29.8%–37.9%) of them utilized health institutions for their last delivery. History of still birth (AOR (adjusted odds ratio) =0.25, 95% CI (confidence interval) =0.07–0.77), number of ANC visit (AOR = 38.51, 95% CI = 22.35–66.33), functional media (AOR = 2.61, 95% CI = 1.59–4.28) and distance to nearby health facility (AOR = 0.52, 95% CI = 0.32–0.83) were found to be significantly associated with institutional delivery service utilization.

**Conclusion:**

In this research the level of institutional delivery service utilization is still low compared to government initiatives. History of still birth, low number of ANC visit, unavailability of functional media and existence of distant health facilities were found to be significantly associated with low utilization of the service. So, concerned bodies should contribute their share to improve institutional delivery service utilization in the study area by tackling modifiable risk factors.

## Plain English summary

Institutional delivery service utilization is one of the most important interventions to reduce maternal death. In this study the factors that underlie institutional delivery service utilization was investigated.

To achieve the objective, community based quantitative cross-sectional study was conducted among systematically selected mothers using interviewer administered questionnaire. Descriptive and analytical statistics were applied for the analysis.

One third of study participates were utilized health institutions for their last delivery which is still low compared to government initiatives. Institutional delivery service utilization was determined by history of still birth, number of ANC visit, functional media and distance to nearby health facility. So, concerned bodies should contribute their share to improve institutional delivery service utilization in the study area and tackle modifiable risk factors that affect utilization of the service.

## Background

More than 20 million women worldwide become pregnant annually, 15% of whom are likely to develop complications [1]. The risk of death from complications relating to pregnancy and childbirth over the course of a woman’s lifetime is higher in the developing compared to the developed world which is one in 76, and one in 8000 respectively [2, 3]. Almost all annual deaths from pregnancy related complications are occurring in Asia and Africa, leading the world in pregnancy related complications [3–5]. Most Sub- Saharan African (SSA) countries are not also on track for meeting the targets pertaining to maternal mortality, as recent estimates suggest that the average annual rate of reduction in maternal mortality rate (MMR) in SSA countries is less than 1% [5, 6].

Institutional delivery service utilization is one of the most important interventions to reduce maternal death and the proportion of women who delivered with the assistance of a skilled birth attendant is one of the indicators in every country health plans [7–10]. Maternal death is highest among countries which have less skilled professionals such as trained midwife, Nurse, doctor or other trained health professionals [2]. In almost all countries where health professionals attend more than 80% of deliveries, MMR is below 200 per 100,000 live births [5]. A woman who is going to give birth at a health facility can get sufficient medical care during child birth which in turn can highly prevent maternal and neonatal deaths [10]. However, rate of delivery attended by skilled birth attendants at health facilities is still very low in many developing countries and is lowest in sub-Saharan Africa (average facility delivery in 2008 was only 47%) [11–13].

Being one of the developing countries, pregnancy related complications are among the many health challenges of Ethiopia. The MMR in the country was 676 per 100,000 live births in 2011 which was not significantly different from the 2005 EDHS report (673 per 100,000 live births) [14, 15]. Major causes of maternal deaths in Ethiopia are preventable that include hemorrhage (APH and PPH), prolonged/obstructed labor and ruptured uterus, severe pre-eclampsia and eclampsia, sepsis, and complications of abortion which account for 69% of the deaths [16]. This high maternal death are due mainly to the fact that over 90% of births take place at home, and women with complications may not be arriving at a health facility in time (Federal ministry of health (FMOH): National Baseline Assessment for Emergency Obstetric and Newborn Care in Ethiopia, unpublished). Even though the country is aggressively constructing and equipping health facilities with staff and equipment’s, and strategies like user-fee exemption for delivery and associated cares have been in place to enhance access, still the use of health facilities for maternal health services is very low. According to 2011 EDHS, skilled delivery attendance in Ethiopia was only 10% of the total deliveries [14]. For the observed low coverage different barriers are incriminated that are varying in different contexts [17–25]. Literatures from different corners of the country are also show discrepancies in findings indicates the importance of site specific studies on it especially in areas where access to health information is limited. Though there are literatures in Ethiopia on level and factors associated with institutional delivery, little is known about the issue in the study area. Investigating the issue provides an opportunity for different stakeholders and health workers to tackle factors that could possibly influence institutional delivery service utilization. Therefore, the objective of this study was to assess factors influencing institutional delivery service utilization among mothers who gave birth within the last two years in Dembecha district, Northwest Ethiopia from March 1 to 30, 2015.

## Methods

### Study design and period

A community based cross-sectional study was carried out from March 1 to March 30, 2015 among 700 randomly selected pregnant women who lived in the selected Kebeles of the district for more than six months.

### Study area

The study was conducted in Dembecha district, West Gojam zone, Amhara region located at the north western part of Ethiopia, about 350 km from Addis Ababa and 220 km from Bahir Dar. The district has four urban 25 rural Kebeles (lowest administration unit in Ethiopia). Within the district there are six health centers and 25 health posts. According to Dembecha district health office report, 2015 population projection estimate, Dembecha district has total population of 129,977 of which 66,475 are female and 31,720 of the total female population are in a reproductive age group (15–49 years). The total number of women deliver per year was expected to be 7376 with general fertility rate of 232.5/1000 [26].

### Sample size and sampling procedure

The sample size was calculated using a single population proportion formula with the following assumptions: P = Expected proportion of the population (proportion of institutional delivery service utilization is 15.7%) [22], d = Desired precision/the margin of error (4%), a design effect of two (as stratified multistage sampling was used) and 10 % non-response rate. Accordingly the required, sample size was 700. Since this research considers institutional delivery as deliveries attended either at hospitals, health centers or health posts, there is heterogeneity in service delivery across urban and rural Kebeles. Therefore, this study utilized multi stage sampling with stratification sampling technique to select the study participants. The district was classified into urban and rural strata. Kebeles (one urban and six rural) and study participants (700 women) from each stratum were selected using lottery and systematic sampling methods respectively. Sampling frame (list of all women that gives birth within last two years from each selected Kebeles) was obtained from Health extension workers (HEWs) in the selected Kebeles since they record all mothers in their location regularly to prepare them for maternal health care services.

### Data collection tool and procedure

Data collection questionnaire was adapted from tools used to assess institutional delivery service utilization used by different studies that could satisfy the objectives of the study and variables under study [6, 14, 17, 19, 20, 22–25]. Semi-structured questionnaire was used and it was translated in to Amharic language (local language) then back to English language to check consistency. The data was collected by eight clinical nurses who were not working in a health facility where the selected kebele is under its catchment)and supervised by two BSc nurses. The data collectors and supervisors were trained for one day on how to interview and how to fill the questionnaire based on a prepared instruction. The tool was pretested seven days before the actual data collection on 5% of the total sample at non-selected Kebele of the study district among women who gave birth within the last two years.

### Data quality assurance

To increase the quality of data; translated and pretested questionnaire was used, data collectors were trained and close supervision was made during the data collection. The collected data were also cross checked on each day of activity for consistency and completeness. Double entry of data and cleaning of data using frequency, sorting and listing to identify any missed value and outlier was made and identified errors was cross checked with the original questionnaire.

### Data processing and analysis

The collected data were double entered using EPI Data software version 3.0. Then it was exported to SPSS version 16.0 for data processing and analysis. The outcome variable, delivery service utilization was dictated as “YES = 1” if a mother gave birth only in hospital, health center or health post for her recent delivery and NO = 0 (women who delivered at home). Descriptive statistics like proportions mean and standard deviations and analytic tests like bivariate and multivariable logistic regression analysis were computed. Odds ratio along with the 95% CI was estimated to ascertain the association between covariates and institutional delivery service utilization. Covariates that have *P*-value of <0.2 at the bivariate analysis were included in the multivariable logistic regression to control all possible confounding factors. For all statistical tests *P*-value <0.05 was used as a cut-off point for statistical significance.

### Ethical consideration

Ethical clearance was obtained from research committee of Debre Markos University, College of medicine and health sciences. The heads of the district health office were informed about the purpose of the study and permission was obtained. Informed consent was also obtained from study participants. All information obtained from the study participants was kept confidential and at the end of the interview mothers with deliveries other than health institutions were advised to use health facilities for obstetric care services for the next planned pregnancy.

## Results

### Socio-demographic characteristics of respondents

A total of 674 out of 700 women who gave birth within the last two years were interviewed with a response rate of 96.3%. The 26 non-responders were due to migration (one), absent after three time’s visit (nine) and refusal to participate (sixteen). The mean age of the study participants was 31.93 ± 6.2. Of the total respondents, majority of them were rural residents, 577(85.6%), Orthodox in their religion 659(97.8%), married, 641 (95.1%) and house wives, 525(77.9%). Most of respondent’ husbands’ were farmers 497 (77.5%). Regarding to educational status, majority of women and their husband were able to read and write but without formal education, 280 (41.5%) and 272 (42.4%) respectively (Table 1).

**Table 1:** Socio-demographic characteristics of respondents, Dembecha district, Northwest Ethiopia, 2015 (*n* = 674)

Variable	Category	Frequency	percent
Residence	Urban	97	14.4
	Rural	577	85.6
Age	15–19 years	8	1.2
	20–24 years	51	7.6
	25–29 years	202	30.0
	30–34 years	173	25.7
	35+ years	240	35.6
Educational status of a mother	Unable to read & write	256	38.0
	Able to read & write	280	41.5
	Primary education (1–8)	109	16.2
	Secondary and above (9–12+)	29	4.3
Educational status of a husband (*n* = 641)	Unable to read & write	108	16.8
	Able to read & write	272	42.4
	Primary education (1–8)	196	30.6
	Secondary and above (9–12+)	65	10.1
Religion	Orthodox	659	97.8
	Other	15	2.2
Marital status	Married	641	95.1
	Divorced	18	2.7
	single	13	1.9
	Widowed	2	0.3
Mother’s occupation	House wife	525	77.9
	Employed/Merchant	86	12.8
	Daily laborer/Students	57	8.5
	Others	6	0.9
Husband’s occupation	Farmer	497	77.5
	Employed/Merchant	98	14.5
	Daily laborer/Students	45	6.7
	Others	1	0.2
Size of a family	1	37	5.5
	2–3	148	22
	4–5	275	40.8
	>5	214	31.7

### Obstetric characteristics of respondents

Majority of study participant’s age at first marriage 334 (49.6%) and age at first pregnancy 381 (56.5%) was 15–19 years. Most 421 (62.5%) and 445 (66%) had two to four history of gravidity and parity respectively but 87 (12.4%) and 53 (7.6%) had a history of abortion and still birth in their life time. 511 (75.8%) of the deliveries were planned pregnancies. Out of the total pregnancies, 311 (46.1%) of them had ANC visits. Among pregnancies which had ANC visits, 179 (56.8%) had two to three ANC visits (Table 2).

**Table 2:** Obstetric characteristics of respondents, Dembecha district, Northwest Ethiopia, 2015 (*n* = 674)

Variable	Category	Frequency	percent
Age at first marriage	<15 years	197	29.2
	15–19 years	334	49.6
	20–24 years	122	18.1
	25+ years	21	3.1
Age at first pregnancy	<15 years	4	0.6
	15–19 years	381	56.5
	20–24 years	242	35.9
	25+ years	47	7
Gravidity	1	96	14.2
	2–4	421	62.5
	≥5	157	23.3
Parity	1	105	15.6
	2–4	445	66
	≥5	124	18.4
Abortion in life time	Yes	86	12.8
	No	588	87.2
Still birth in life time	Yes	51	7.6
	No	623	92.4
Planned pregnancy	Yes	511	75.8
	No	163	24.2
ANC visit	Yes	311	46.1
	No	363	53.9
Number of ANC visits(*N* = 311)	Only one	54	17.4
	Two-three	179	57.6
	Four and above	78	25.1

### Accessibility characteristics of respondents

Most of respondents has health facility within one to two hour distance, 374 (55.5%) and have available functional media, 433 (64.2%) (Table 3).

**Table 3:** Accessibility characteristics of respondents, Dembecha district, Northwest Ethiopia, 2015 (*n* = 674)

Variable	Category	Frequency	Percent
Distance to nearby Health institution	<1 h	285	42.3
	1–2 h	374	55.5
	>2 h	15	2.2
Availability of functional media	Yes	433	64.2
	No	241	35.8

### Level of institutional delivery utilization

**Fig. 1:**
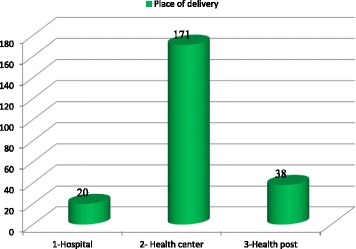
Distribution of institutional delivery service utilization by facility, Dembecha district, Northwest Ethiopia, 2015 (*n* = 229)

Out of 674 interviewed mothers who gave birth within the last two years, 229 (34%; 95% CI: 29.8%–37.9%) of them utilized health institutions for their last delivery and the rest, 445 (66%) were delivered at home. Among mothers who utilized health institutions for their last delivery, their most visited place of delivery was health center 171 (74.7%) (Fig. 1).

### Factors associated with institutional delivery service utilization

The results of bivariate and multivariable analysis between institutional delivery service utilization and selected independent factors are presented in Table 4. Accordingly, in the bivariate analysis; educational status of women, marital status, family size, gravidity, abortion history, still birth history, planned pregnancy, ANC visit, presence of functional media and distance from nearby health facility were the main significant factors associated with institutional delivery service utilization (Table 4).

**Table 4:** Factors independently associated with institutional delivery service utilization, Dembecha district, Northwest Ethiopia, 2015 (*n* = 674)

Variable	Institutional delivery service utilization		COR	AOR	
	No	Yes	(95% CI)	(95% CI)	*P*-value
Place of residence					
Urban	71	26	1.00		
Rural	374	203	1.48 (0.91–2.39)		
Age (years)					
15–19	4	4	1.00		
20–24	27	24	0.88 (0.20–3.94)		
25–29	111	91	0.82 (0.19–3.36)		
30–34	112	61	0.54 (0.13–2.25)		
35+	191	49	0.25 (0.06–1.06)		
Educational status					
Unable to read & write	192	65	1.00		
Able to read & write	175	104	**1.75 (1.21–2.54)**		
Primary education (1–8)	62	47	**2.23 (1.39–3.59)**		
Secondary and above (9–12+)	16	13	**2.40 (1.09–5.52)**		
Current marital status					
Married	416	225	1.00		
Unmarried	29	4	**0.25 (0.08–0.73)**		
Gravidity					
1	44	52	1.00		
2–4	283	138	**0.41 (0.26–0.64)**		
≥ 5	118	39	**0.28 (0.16–0.48)**		
Abortion in life time					
No	380	208	1.00		
Yes	65	21	**0.59 (0.35–0.99)**		
Still birth in life time					
No	400	223	1.00	1.00	
Yes	45	6	**0.24 (0.10–0.57)**	**0.25 (0.07–0.77)**	0.016
Planned pregnancy					
No	125	38	1.00		
Yes	320	191	**1.96 (1.31–2.94)**		
ANC visit					
No	341	22	1.00	1.00	
Yes	104	207	**30.85 (18.87–50.42)**	**38.51 (22.35–66.33)**	0.0001
Distance to health facility					
< 1 h	172	113	1.00	1.00	
1–2 h	273	116	**0.64 (0.46–0.89)**	**0.52 (0.32–0.83)**	0.006
Available functional media					
No	192	49	1.00	1.00	
Yes	253	180	**2.78 (1.93–4.02)**	**2.61 (1.59–4.28)**	0.0001

*COR* crude odds ratio, *CI C*onfidence interval, *AOR* adjusted odds ratio

Bold cases indicate significance

In the final model (multivariable analysis); variables with *P*-value of >0.2, and parity and family size that show multicollinarity with gravidity were not included. Whereas other variables like: place of residence, age, educational status of a mother, marital status, family size, gravidity, abortion history, still birth history, planned pregnancy, ANC visit, presence of functional media and distance from nearby health facility were found to have *P*-value <0.2 in which this variables were taken to multivariable analysis. Accordingly; the results of the final multiple variable logistic regression model revealed history of still birth, number of ANC visit, functional media and distance to nearby health facility were found to be significantly associated with institutional delivery service utilization in multivariable analysis with *P*-value <0.05 (Table 4).

## Discussion

In this study the level of institutional service utilization was 34%. This finding is lower than the findings from India 95.2% and 84.9% [27, 28]; Tanzania 74.5% [29]; Ghana 37.5% [30] and Bahir Dar, Ethiopia 78.8% [17] and Woldeia, Ethiopia 48.3% [23]. But higher than findings from Munisa Woreda, South East Ethiopia 12.3% [18]; Sekela District, North West of Ethiopia, 12.1% [19]; Dodota district, Oromia region, Ethiopia 18.2% [20]; Rural Jimma Horro District, Southwest Ethiopia 8% [21]; Banja District, Awi Zone, Amhara Region, Ethiopia 15.7% [22] and mini-EDHS 2014 report, 12% in Amhara Region and 16% in Ethiopia [15]. The possible explanations for the observed differences might be related with differences in study place, year of study, sample size and study design. Another possible explanation could be introduction of the new “mother waiting center approach” in the study area that create opportunity for mothers to deliver at health institution.

In this study; the association of respondent’s and their partner’s socio-demographic variables, obstetric variables and accessibility variables with institutional delivery service utilization was assessed. Those mothers having history of ANC visit were 38 times more likely (AOR = 38.51, 95% CI = 22.35–66.33) to utilize health institutions for delivery compared to those who had no history of ANC visit. This finding was supported by findings from Tanzania [29], Ghana [30], Nigeria [31] and Ethiopia [17–19]. This might be due to the fact that among ANC services, birth preparedness plan is a major task at ANC4 and majority of mothers might get convinced to deliver at health institutions.

On the other hand, those mothers having a history of still birth in life time were 75% less likely (AOR =0.25, 95% CI = 0.07–0.77) to utilize health institutions for delivery compared to those who had no a history of still birth. The possible explanations for this finding could be some of the mothers with a history of still birth may be a habit of delivering at home, and may develop attitude of ignorance hence be ambivalence in delivering at health institutions.

Those mothers who were living in a place where 1–2 h distance away from nearby Health institution were 48% more likely (AOR = 0.52, 95% CI = 0.32–0.83) to utilize health institutions for delivery compared to those who were living in a place where less than 1 h distance away from nearby Health institution. This finding was also supported by other findings from Ethiopia [19, 21, 24]. This is because mothers who were living near health institutions may have access to health education, ANC service and transport.

Mothers who had functional media were two and half more likely (AOR = 2.61, 95% CI = 1.59–4.28) to utilize health institutions for delivery compared to those who had no functional media. The possible explanation for this finding is mothers who had access to media may have a chance to get informed about institutional delivery service and consequences of home delivery.

### Limitations of the study

Data was collected from mothers about their experience since 2 years that might lead to a recall bias and cross sectional nature of the study could not allow cause effect relationship.

## Conclusions

In this research the level of institutional delivery service utilization is one in every three women which is still low compared to government initiatives which is increasing to 60% the proportion of births attended by skilled health personnel either at home or in a facility. Still birth history, ANC visit, availability of functional media and distance from health facility were found to be significantly associated with institutional delivery service utilization. Based on the findings the following recommendations are forwarded:

- Focused ANC and mother waiting centers for delivery service need to be strengthened.
- Transportation and Ambulance services need to be available and accessible so that mothers can access the health facilities in a short period of time.
- Awareness creation needs to be done about birth preparedness plan for pregnant mothers during their ANC visit.
- Health professionals working in the district should follow “mother friendly approach” in providing maternal health services and need to create opportunities mothers to share experiences about using health institutions for delivery service
